# Role of Astrocytes in Central Respiratory Control

**DOI:** 10.3390/cells15161447

**Published:** 2026-08-11

**Authors:** Yasumasa Okada, Isato Fukushi, Shigefumi Yokota, Kotaro Takeda, Akira Umeda, Mieczyslaw Pokorski, Hiroshi Onimaru

**Affiliations:** 1 Clinical Research Center, Murayama Medical Center, 2-37-1 Gakuen, Musashimurayama, Tokyo 208-0011, Japan; 2 Graduate School of Health Sciences, Aomori University of Health and Welfare, Aomori 030-8505, Japan; 3 Department of Anatomy, Faculty of Medicine, Tottori University, Yonago 683-8503, Japan; 4 Faculty of Rehabilitation, School of Health Sciences, Fujita Health University, Toyoake 470-1192, Japan; 5 Department of Respiratory Medicine, International University of Health and Welfare Shioya Hospital, Yaita 329-2145, Japan; 6 Institute of Health Sciences, University of Opole, 45-040 Opole, Poland; 7 Department of Physiology, Showa Medical University School of Medicine, Tokyo 142-8666, Japan

**Keywords:** astrocyte, glia, hypercapnic ventilatory response, hypoxic ventilatory response, neuroglia, respiratory control, respiratory plasticity, respiratory rhythm generation

## Abstract

Astrocytes, once regarded primarily as structural and metabolic support cells, are now increasingly recognized as active participants in neural information processing. Within respiratory control networks, astrocytes are widely distributed throughout the brainstem and spinal cord, where they engage in bidirectional communication with neurons to regulate breathing. This review summarizes current knowledge regarding the roles of astrocytes in respiratory rhythm and pattern generation, central respiratory chemoreception, hypoxic ventilatory responses, respiratory plasticity, and respiratory pathophysiology. Recent advancements in calcium imaging, optogenetics, and pharmacology have revealed that astrocytes modulate respiratory network activity through intracellular Ca^2+^ signaling and the release of gliotransmitters, particularly ATP. In the ventrolateral medulla and the parafacial respiratory group/retrotrapezoid nucleus, astrocytes contribute to central CO_2_/H^+^ chemoreception through mechanisms involving connexin hemichannels, potassium channels, and purinergic signaling. Emerging evidence further suggests that astrocytes participate in central hypoxic responses and adaptive respiratory plasticity. In addition, astrocytic dysfunction has been implicated in several disorders affecting respiratory control, including brainstem astrocytoma, Rett syndrome, sudden infant death syndrome, and sudden unexpected death in epilepsy. Collectively, accumulating evidence identifies astrocytes as integral components of respiratory control networks that contribute to both the maintenance of respiratory homeostasis and the pathogenesis of respiratory dysfunction. A deeper understanding of astrocyte–neuron interactions may provide novel therapeutic opportunities for the treatment of respiratory disorders.

## 1. Introduction

Maintaining oxygen and carbon dioxide homeostasis is essential for life, and the respiratory system plays a central role in this process. Rhythmic breathing is generated automatically by neuronal networks located in the lower brainstem. Astrocytes are present throughout these respiratory regions in numbers comparable to those of neurons. Although traditionally regarded as structural and metabolic support cells, astrocytes are now recognized as active participants in the sensing of environmental changes and the maintenance of neural homeostasis.

Until the early 2000s, astrocytes were generally not considered to play an active role in neural information processing [1,2,3,4,5]. Subsequent studies demonstrated that astrocytes actively participate in a wide range of brain functions through reciprocal communication with neurons [6,7,8]. These findings prompted the hypothesis that astrocytes may also contribute to respiratory control networks by modulating respiratory output in response to physiological demands [9,10,11,12,13,14].

Accumulating evidence has demonstrated that astrocytes influence multiple aspects of respiratory control, including respiratory rhythm and pattern generation, central chemoreception, hypoxic ventilatory responses, and respiratory plasticity. More recently, astrocytic dysfunction has also been implicated in the pathogenesis of several respiratory disorders. Furthermore, phylogenetic studies have shown that astrocytes within the paratrigeminal respiratory group, a proposed respiratory central pattern generator, contribute to respiratory neural output in the lamprey [15,16]. These findings suggest that astrocyte-dependent mechanisms involved in respiratory regulation have been evolutionarily conserved since the earliest stages of vertebrate development.

In this review, we summarize current knowledge regarding the roles of astrocytes in respiratory control under both physiological and pathological conditions and discuss emerging concepts that may provide new insights into respiratory regulation and disease.

## 2. Distribution of Astrocytes in Respiration-Related Regions of the Brainstem and Spinal Cord

Astrocytes are distributed throughout the brainstem; however, their density is particularly high within the superficial rostroventral chemosensitive medullary region, which approximately corresponds to the parafacial respiratory group (pFRG)/retrotrapezoid nucleus (RTN). In this area, astrocytes form a dense layer beneath the ventral medullary surface, referred to as the marginal glial layer or glia limitans [17,18,19]. Beneath this superficial layer, astrocytes are more diffusely distributed throughout the brainstem parenchyma, both within and outside respiration-related regions.

**Figure 1:**
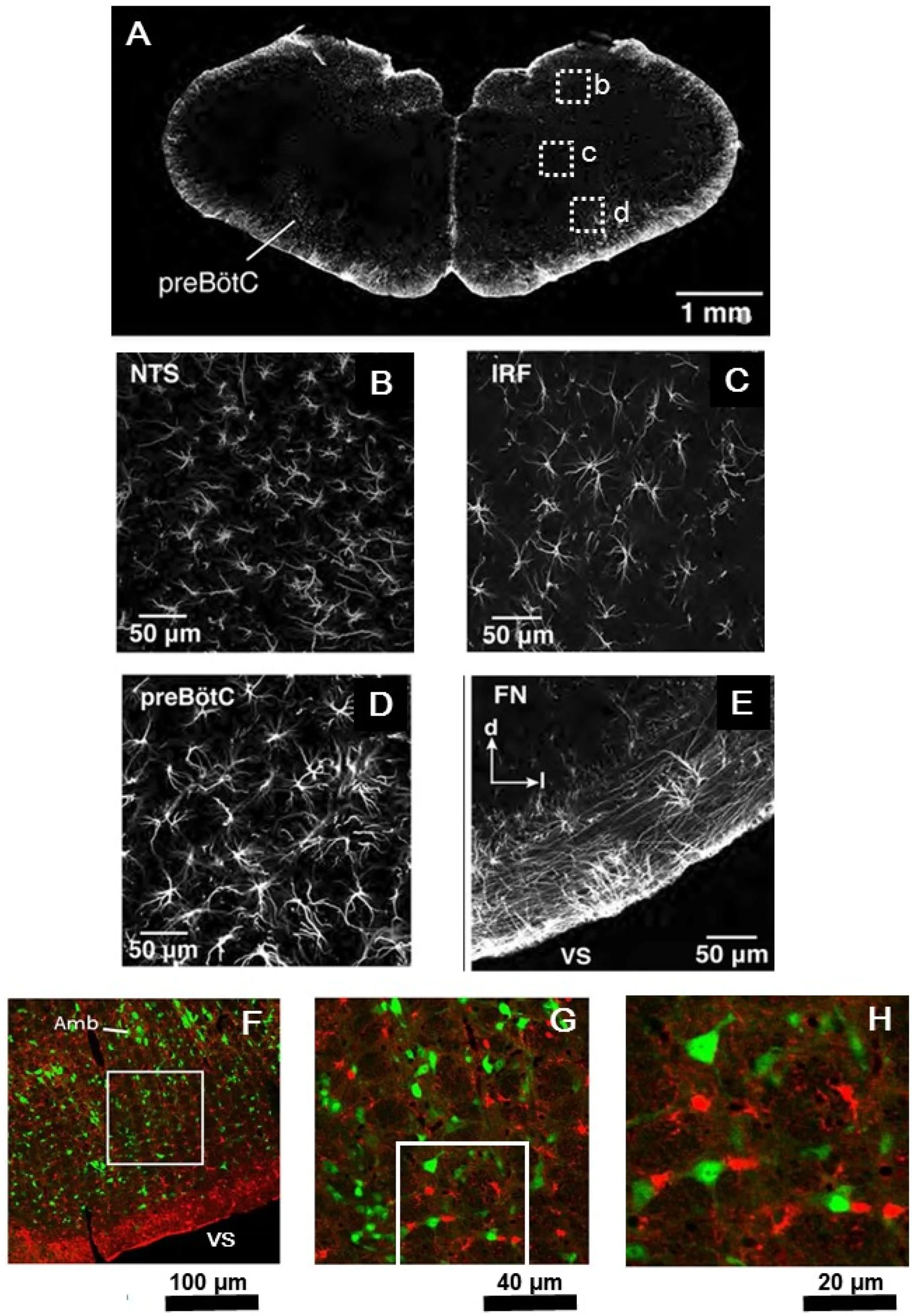
Distribution of astrocytes in the adult rat brainstem. The dorsal side is shown at the top. (**A**). Distribution of astrocytes at the level of the pre-Bötzinger complex (preBötC) in the medulla. Astrocytes were immunolabeled with astrocyte-specific marker GFAP. Dashed squares b, c, and d indicate the regions of the NTS, IRF, and preBötC, which are shown at higher magnification in panels (**B**, **C**, and **D**), respectively. (**E**). A high-magnification image of the retrotrapezoid nucleus (RTN). Astrocytes form a dense network of processes within the marginal glial layer covering the RTN. FN, facial nucleus. VS, ventral surface. (**F**). Spatial relationship between astrocytes and neurons in the rat ventrolateral medulla. Astrocytes (cell bodies) and neurons (nuclei) were identified as astrocyte-specific marker S100-positive cells (red) and neuron-specific marker NeuN-positive cells (green), respectively. The square in panel (**F**) indicates the preBötC region, and corresponds with the area in (**G**). Amb, nucleus ambiguous. (**G**). An enlarged image of the preBötC. The square indicates the area in (**H**). (**H**). A high-magnification image showing the close spatial association between neurons and astrocytes. Adapted from Sheikhbahaei et al. (2018) [19] (panels **A**–**E**) and from Ikeda et al. (2017) [20] (panels **F**–**H**), both under the terms of Creative Commons CC BY 4.0.

Sheikhbahaei et al. (2018) analyzed and compared the morphological characteristics of astrocytes in the nucleus tractus solitarius (NTS), intermediate reticular formation (IRF), and pre-Bötzinger complex (preBötC) of adult rats [19]. They reported that astrocytes in the preBötC exhibit greater morphological complexity, i.e., longer processes, more branch points and terminals, and greater convex hull volume and surface area than those in the NTS and IRF [19]. In addition, astrocytes and neurons within the preBötC are closely intermingled and frequently establish intimate cellular associations (Figure 1) [20]. This morphological complexity of preBötC astrocytes may indicate their role in providing region-specific structural/metabolic support and fulfilling a higher density “tripartite” synaptic function important for the modulation of breathing rhythm.

In the human medulla, astrocyte-rich niches have been identified in several regions, including the superficial layer of the rostral ventrolateral medulla, the median raphe, arcuate nucleus, area postrema, and NTS. Notably, several of these regions have been proposed to function as central respiratory chemoreceptive sites [21].

**Figure 2:**
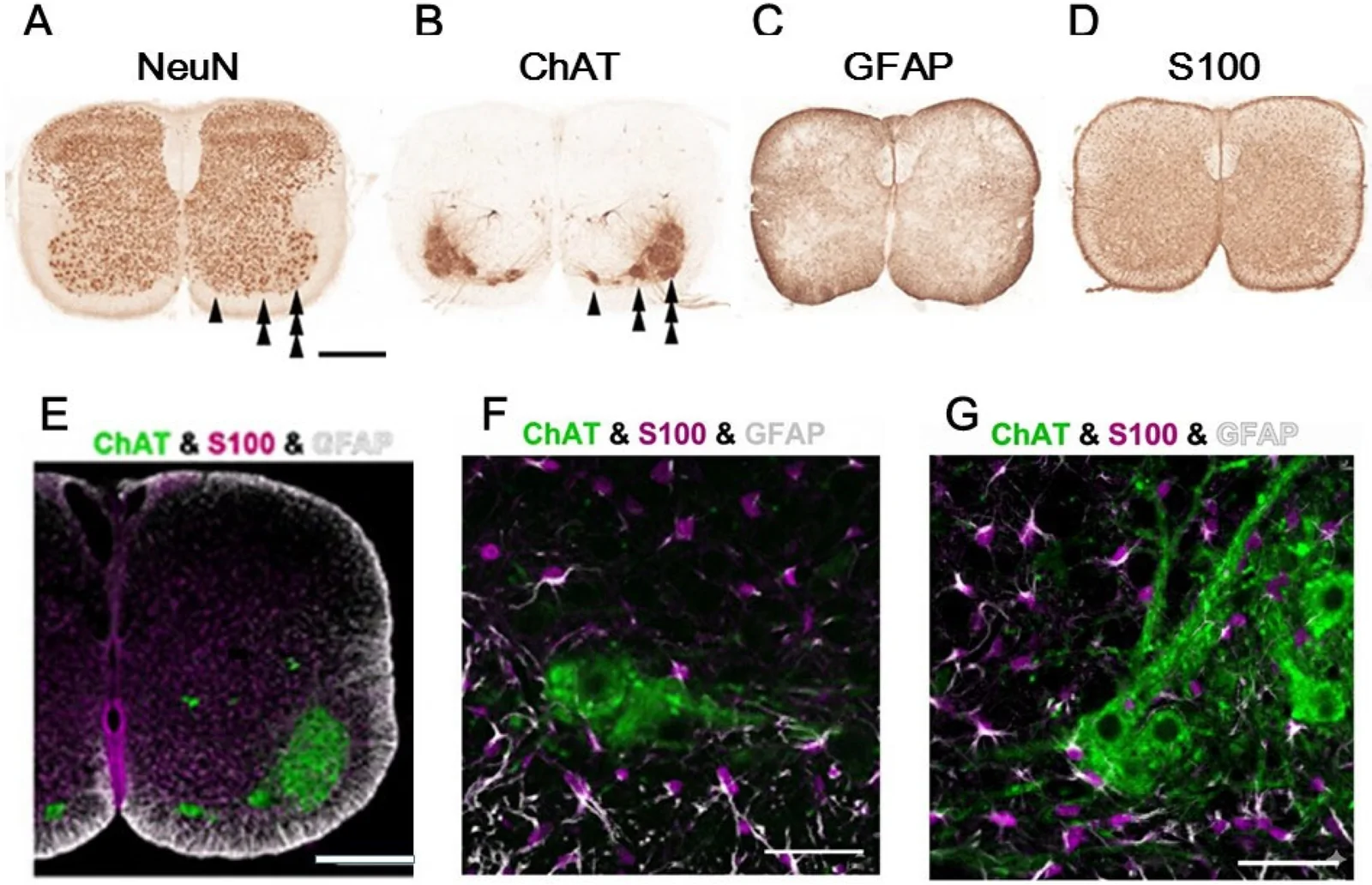
Distribution of astrocytes and neurons at the level of the phrenic motor nucleus of the cervical spinal cord of the rat. (**A**). Immunostaining of neuronal nuclear antigen (NeuN) for neurons. (**B**). Choline acetyltransferase (ChAT)-immunoreactive neurons identifying putative motoneurons. Single-, double-, and triple-arrowheads indicate medial, central, and lateral motor columns, respectively. (**C**). Glial fibrillary acidic protein (GFAP) for astrocytes. (**D**). S100-protein β-subunit (S100) for astrocytes. (**E**). Confocal image showing the distribution of immunoreactivities of ChAT, S100 and GFAP, indicated as green, magenta, and white, respectively, at the C4 level of the cervical spinal cord. (**F**,**G**). High-magnification images of scalene and phrenic motoneuron populations in the medial and central motor regions, respectively, surrounded by astrocytes (S100-ir and GFAP-ir cells). Scale bars, (**A**–**D**), 500 µm; (**E**), 300 µm, (**F**) and (**G**), 50 µm. Reproduced from Shinozaki et al. (2019) under the terms of Creative Commons CC BY 4.0 [22].

At the level of the phrenic motor nucleus in the cervical spinal cord, astrocytes and neurons are broadly distributed throughout the transverse plane of the spinal cord. Phrenic motoneurons, identified by choline acetyltransferase (ChAT) immunoreactivity, are localized exclusively within lamina IX of the ventral horn. Like other motor nuclei, the phrenic motor nucleus, situated within the central motor column of lamina IX, is surrounded by astrocytes (Figure 2) [22].

The close anatomical association between astrocytes and respiratory-related neurons in the brainstem and spinal cord suggests the existence of bidirectional signaling pathways between these cell populations. Such neuron–astrocyte interactions are thought to contribute to the dynamic modulation of respiratory motor circuits and to maintain network activity within an optimal functional range [23,24].

## 3. Role of Astrocytes in the Generation and Modulation of Respiratory Rhythm and Pattern

Rhythmic respiratory activity is generated by brainstem neuronal networks collectively referred to as central pattern generators (CPGs). Classical models of respiratory rhythmogenesis considered these networks to be composed exclusively of interconnected neurons, whose intrinsic membrane properties and synaptic interactions were thought sufficient to account for rhythmic activity. This neuron-centric view has been challenged by growing evidence that astrocytes actively integrate and process synaptic information and can modulate the activity of rhythm-generating neuronal circuits [23,24,25,26,27,28].

Early evidence for astrocytic involvement in respiratory rhythm generation was provided by Hülsmann et al. (2000), who demonstrated that inhibition of glial metabolism with fluoroacetate, a selective inhibitor of the astrocytic Krebs cycle, depressed rhythmic respiratory activity in medullary slices from neonatal mice [1,29]. At the time, astrocytes were considered primarily metabolic support cells, supplying glutamine to glutamatergic neurons through the glutamate–glutamine cycle. Consistent with this interpretation, Young et al. (2005) showed that treatment of neonatal rats with methionine sulfoximine, an inhibitor of astrocytic glutamine synthetase, reduced respiratory frequency and attenuated hypercapnic ventilatory responses [30,31]. Similarly, Li et al. (2010) reported that methionine sulfoximine abolished the respiratory stimulatory effects of doxapram in rhythmically active neonatal rat brainstem slices [32]. Together, these findings indicated that normal astrocytic metabolism is required for the maintenance of respiratory network function. However, when interpreting these data, it must be noted that each of these pharmacological agents has its distinct astrocyte specificity limitation. This issue is further discussed in Section 8.2.

**Figure 3:**
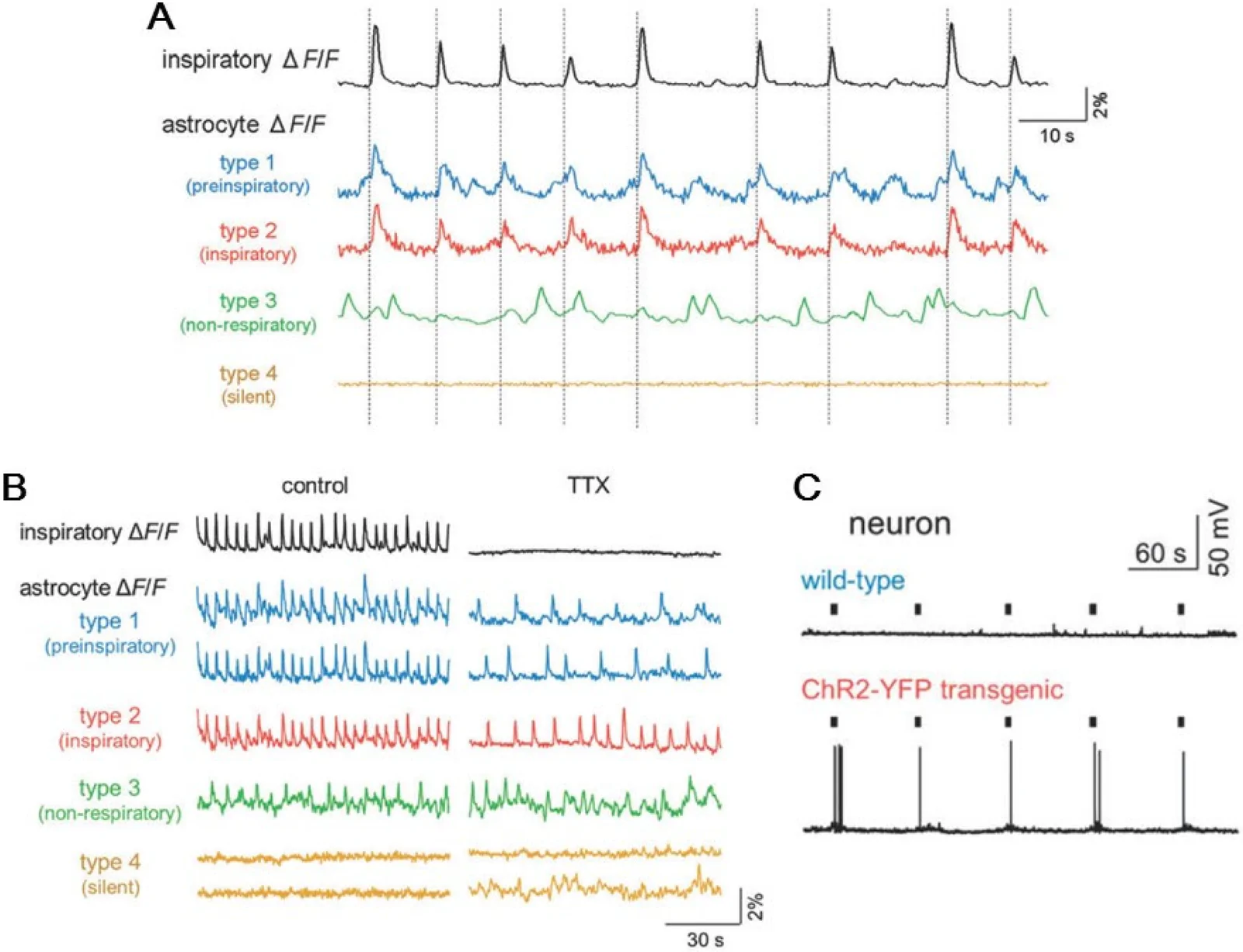
Intrinsically oscillating astrocytes with preinspiratory activity. (**A**). Preinspiratory activity of putative astrocytes. Representative calcium Δ*F*/*F* traces of preinspiratory, inspiratory, non-respiratory, and silent astrocytes. Top: bulk Δ*F*/*F* fluctuations of the preBötC (neuron). (**B**). Intrinsically rhythmic activities of preinspiratory astrocytes. Representative population inspiratory Δ*F*/*F* traces (top) and calcium Δ*F*/*F* traces of preinspiratory, inspiratory, non-respiratory, and silent astrocytes before and after application of tetrodotoxin. Note that the astrocyte classified as silent before tetrodotoxin application exhibits rhythmic activity afterward. (**C**). Photoactivation of astrocytes triggers action potentials in an inspiratory neuron from the preBötC in a ChR2(C128S)YFP transgenic, but not in wild-type mouse. Black bars indicate the activation periods of preBötC astrocytes. Reproduced from Okada et al. (2012) with permission from John Wiley & Sons [35].

These observations raised an important question: do astrocytes merely support respiratory rhythm generation metabolically, or do they actively participate in rhythmogenesis? Although this issue remains debated [26], several studies have provided evidence supporting an active role of astrocytes in respiratory network dynamics. Because astrocytes do not generate action potential, calcium imaging has become a principal approach for investigating astrocytic activity within respiratory circuits [33]. Using functional calcium imaging, Huxtable et al. (2010) demonstrated that astrocytes contribute to purinergic excitation of preBötC rhythm-generating networks [34]. Likewise, we observed rhythmic intracellular calcium oscillations in a subset of preBötC astrocytes that preceded inspiratory neuronal activity in rhythmically active mouse medullary slices (Figure 3A) [35]. Notably, these preinspiratory astrocytes continued to exhibit rhythmic calcium activity during tetrodotoxin-induced neuronal silencing, although oscillation frequency was reduced and synchronization between astrocytes was disrupted (Figure 3B) [35]. Furthermore, optogenetic activation of preBötC astrocytes elicited firing of inspiratory neurons (Figure 3C) [35]. It must be noted that this temporal association, i.e., activity of preinspiratory astrocytes precedes neuronal bursts, does not prove that these astrocytes initiate respiratory rhythm. Although we propose that a subset of preBötC astrocytes possesses intrinsic oscillatory properties that enable them to generate respiratory rhythm, this idea remains a hypothesis and requires rigorous experimental validation [35].

Subsequent studies have produced mixed results regarding the extent of astrocytic participation in respiratory rhythmogenesis. Schnell et al. (2011) reported an absence of respiration-synchronized astrocytic activity in medullary respiratory regions [36]. However, reanalysis of their dataset by Oku et al. (2016) identified respiratory phase-related astrocytic activity [37]. Additional studies using rhythmic mouse slice preparation also detected respiration-synchronized calcium activity in subsets of astrocytes [38,39] (Suppl. Figure 2D), [40] (Suppl. Figure 2). In contrast, Tacke et al. (2023), using a working heart–brainstem preparation and fiber photometry, failed to detect astrocytic calcium signals synchronized with inspiratory phrenic bursts [41]. These discrepancies may reflect methodological differences, preparation-dependent effects, or functional heterogeneity among astrocyte populations. Therefore, results obtained from experiments using different types of preparations and different recording techniques cannot be equivalently evaluated. For example, while neonatal slices allow high-resolution cellular imaging, they lack mature intact network connectivity, peripheral inputs, and normal physiological temperature/oxygenation, which may artificially unmask or alter astrocytic intrinsic oscillations. Conversely, while the working heart-brainstem preparation preserves a more intact network, fiber photometry used in that study provides a bulk population signal that may fail to detect low-amplitude, desynchronized, or microdomain-specific calcium transients in a small subset of respiratory astrocytes. These methodological limitation issues are also summarized and discussed in Section 8.

Recent studies have further implicated astrocytes in the generation and modulation of non-eupneic respiratory behaviors, particularly sighs. Severs et al. (2023) demonstrated that pharmacologically induced elevation of astrocytic intracellular calcium increased sigh frequency in mouse slice preparations [42]. Although spontaneous astrocytic calcium transients were only weakly correlated with naturally occurring sighs, optogenetic activation of preBötC astrocytes reliably evoked sigh-like activity through a P2Y_1_ receptor-dependent mechanism. Similarly, Zhao et al. (2025) used in vivo fiber photometry to show enhanced astrocytic calcium signaling associated with sighs and transient respiratory augmentation, although not with baseline eupneic breathing [43]. Optogenetic stimulation of astrocytes significantly increased respiratory frequency in anesthetized mice. More recently, Oliveira et al. (2026) reported that a distinct population of ventrolateral medullary astrocytes is activated before and during sigh generation and is recruited by hypoxia, suggesting a role in coordinating respiratory and arousal responses to oxygen deprivation [44].

Evidence supporting astrocytic participation in rhythm-generating motor networks is not limited to respiration. In the trigeminal masticatory system, Morquette et al. (2012, 2015) demonstrated that rhythmogenesis depends in part on astrocyte-mediated regulation of extracellular calcium concentrations, which modulates the excitability of rhythmogenic neurons [45,46]. These findings raise the possibility that astrocyte-dependent modulation represents a general organizational principle shared among vertebrate central pattern-generating networks.

Additional support for an active role of astrocytes in respiratory control was provided by Sheikhbahaei et al. (2018), who showed that selective blockade of vesicular gliotransmitter release from preBötC astrocytes reduced respiratory frequency, attenuated ventilatory responses to hypoxia and hypercapnia, and decreased exercise capacity [47]. Likewise, Bishop and Sheikhbahaei (2024) demonstrated that suppression of astrocytic signaling in conscious animals slowed breathing and reduced arousal, whereas enhanced astrocytic purinergic signaling increased respiratory activity and behavioral arousal [48]. More recently, Zhao et al. (2025) identified an astrocyte-to-neuron signaling pathway involving connexin43 (Cx43)-dependent ATP release and activation of neuronal P2Y_1_ receptors within the preBötC [43]. Disruption of this pathway altered respiratory motor output and destabilized breathing patterns, highlighting the importance of astrocytic signaling in respiratory network stability.

Cumulatively, current evidence strongly supports the view that astrocytes are active components of respiratory rhythm-generating networks. Nevertheless, whether astrocytes directly generate respiratory rhythms or primarily modulate neuronal rhythmogenic mechanisms remains unresolved. Clarifying the precise contribution of astrocytes to eupneic breathing, sigh generation, and gasping continues to represent a major challenge in respiratory neurobiology.

A major mechanism through which astrocytes influence respiratory circuits is the release of gliotransmitters. Among these, ATP has emerged as the most extensively studied mediator of astrocyte–neuron communication within respiratory control networks. Once released, ATP acts on purinergic receptors expressed by neighboring neurons and glial cells, thereby modulating respiratory network excitability and motor output. Evidence supporting a role for ATP-mediated astrocyte–neuron signaling in respiratory rhythm generation has accumulated steadily over the past decade. Huxtable et al. (2010) demonstrated that ATP signaling contributes to excitatory modulation of preBötC respiratory networks [34]. Subsequent studies showed that selective activation of astrocytes within respiratory regions increases extracellular ATP concentrations and enhances respiratory activity, whereas inhibition of purinergic signaling attenuates these effects [49,50,51].

In addition to ATP, astrocytes release a variety of signaling molecules capable of influencing respiratory circuits, including glutamate, D-serine, lactate, prostaglandins, adenosine, serotonin, substance P, and thyrotropin-releasing hormone. Many of them stimulate respiratory neurons and modulate astrocytic calcium signals in an autocrine manner [52]. The relative contribution of these mediators remains incompletely understood and may vary among respiratory nuclei and physiological states. Proteinase-activated receptor 1 (PAR1) is essential for brain information processing, as a key mediator of astrocyte-neuron communication. PAR1 is highly expressed in astrocytes and drives astrocyte-mediated synaptic regulation. Huda et al. (2018) reported that activation of PAR1 in NTS astrocytes potentiates presynaptic glutamate release on NTS neurons [53]. This potentiation is mediated by a TRPV1-dependent mechanism, where astrocytes release endovanilloid-like molecules to activate presynaptic TRPV1 channels. The activation of the astrocytic PAR1 pathway excites NTS neurons that send projections to respiratory premotor neurons, resulting in the inhibition of respiratory activity [53]. Based on experiments using brainstem-spinal cord preparations, we found that stimulation of astrocytic PAR1 receptors reduces respiratory frequency [39]. This inhibitory effect is associated with transient hyperpolarization of respiratory-related neurons within the ventrolateral medulla. Our findings suggest that this astrocyte-mediated depression of breathing is partially driven by adenosine [39]. Because extracellular ATP is rapidly degraded to adenosine by ectonucleotidases, purinergic signaling frequently evolves into adenosinergic signaling. Adenosine generally exerts inhibitory effects on respiratory neurons through activation of A1 receptors, thereby providing a potential negative-feedback mechanism that limits excessive network excitation. This dual ATP–adenosine signaling pathway may contribute to the stabilization of respiratory network activity under changing physiological conditions [49].

Despite substantial progress, several fundamental questions remain unresolved. The precise mechanisms underlying gliotransmitter release in vivo, the identity of the most physiologically relevant signaling molecules, and the extent to which astrocytic signaling contributes to respiratory rhythmogenesis versus network modulation continue to be actively debated. Moreover, methodological differences among calcium imaging, optogenetic, chemogenetic, and electrophysiological studies have sometimes yielded conflicting results. Future investigations combining cell-type-specific manipulation with high-resolution in vivo recording approaches will be essential for clarifying the contribution of astrocytes to respiratory network function.

Collectively, available evidence indicates that astrocytes are not passive supporting cells but active regulators of respiratory neural circuits. Through calcium-dependent gliotransmitter release and reciprocal communication with neurons, astrocytes contribute to the modulation, stabilization, and adaptive regulation of respiratory rhythm and pattern generation.

## 4. Role of Astrocytes in Central Respiratory Chemoreception

### 4.1. Are Astrocytes Central Chemoreceptors?

Central respiratory chemoreception is a fundamental homeostatic mechanism that adjusts ventilation in response to changes in arterial CO_2_ and brain extracellular pH. For many decades, central respiratory chemoreception was considered an exclusively neuronal function. Chemosensitive neurons located within the ventrolateral medulla, particularly in the pFRG/RTN, have been firmly confirmed to serve as the primary chemoreceptor cells detecting changes in arterial PCO_2_ and brain extracellular pH [54]. In pFRG/RTN neurons, the chemosensitive mechanisms have been well studied, and the intrinsic H^+^ sensing molecules, i.e., two-pore domain potassium channel TASK-2 and G-protein coupled receptor GPR4, were identified [54].

On the other hand, accumulating evidence over the past several decades suggests that astrocytes also play a significant role in central chemoreception. The earliest observations supporting this possibility can be traced to the pioneering studies of Fukuda and Honda (1975), who demonstrated H^+^-induced depolarization of electrically silent cells located in the ventral medullary surface layer [55]. Although these cells were not identified as astrocytes and were not considered primary chemoreceptors at the time, the findings provided the first indication that non-neuronal elements within chemosensitive regions respond to changes in local acid–base conditions. Subsequent experiments showed that blockade of neuronal synaptic transmission abolished these responses [56], reinforcing the prevailing view that neuronal mechanisms dominated central chemoreception.

Interest in astrocytic participation in chemoreception was renewed by studies from Erlichman and colleagues. Local disruption of astrocytic function within the RTN using fluorocitrate altered tissue pH and respiratory activity, indicating that astrocytes play an important role in regulating the chemosensory microenvironment [57,58,59]. These findings suggested that astrocytes contribute to central chemoreception by influencing extracellular pH and, consequently, the activity of neighboring chemosensitive neurons. Erlichman and colleagues, however, viewed astrocytes as modulators of neuronal chemoreception rather than primary chemosensory cells [60,61,62,63].

**Figure 4:**
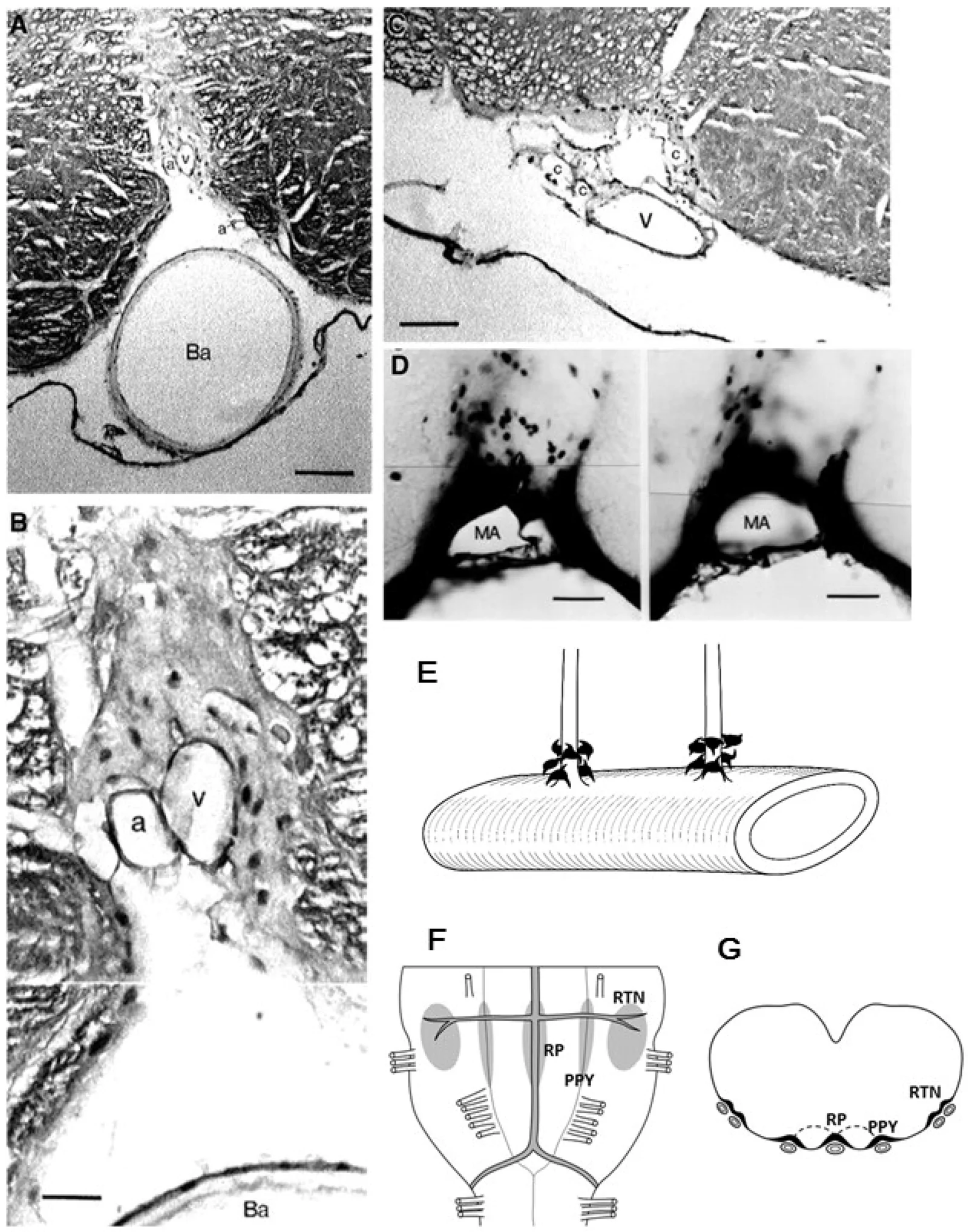
The cell architecture of the central chemoreceptor, composed of astrocyte-like small cells, neurons, and vessels. (**A**–**D**). Photomicrographs of hypercapnia-induced c-fos-positive cell nuclei closely associated with surface vessels in the superficial ventral medulla. (**A**). Clusters of c-fos-positive cells surrounding small vessels (shown as “a” and “v”) in the most ventral portion of the raphe pallidus. The medullary surface of this region is deeply indented and is covered by the basilar artery (Ba). Scale bar, 160 µm. (**B**). Enlarged photograph of (**A**). Hypercapnia-induced c-fos-positive cells are embedded in the homogenous tissue (i.e., thickened marginal glial layer) that is separated from adjacent tissue. Scale bar, 40 µm. (**C**). Clusters of c-fos-positive cells embedded in the thickened part of the marginal glial layer of the parapyramidal region. The pyramidal tract is shown on the right of the photograph. Also in this region, the medullary surface displayed a concave shape and is covered by the petrosal sinus (V) and fine vessels (c). Scale bar, 160 µm. (**D**). Clusters of c-fos positive cells surrounding a penetrating artery [medial medullary artery (MA)] near the medullary surface. Images acquired at two focal planes are shown. Scale bar, 60 µm. Reproduced from Okada et al. (2002) with permission from the American Physiological Society [18]. (**E**). The cell-vessel architecture model for the central respiratory chemoreceptor. Primary chemoreceptor cells (shown in black) surround superficial parts of fine vessels that penetrate the medulla. Primary chemoreceptor cells are located beneath a large surface vessel. Reproduced from Okada et al. (2006) with permission from Springer Nature [64]. (**F**). Localization of central chemoreceptive regions on the ventral medullary surface. They are located mainly in the retrotrapezoid nucleus (RTN), nucleus parapyramidalis superficialis (PPY), and nucleus raphe pallidus (RP). (**G**). Sites of chemoreceptive regions shown on a transverse plane at the rostral medullary level. They share a common anatomical feature in RTN, PPY, and RP; the medullary surface portions are covered, compressed, indented by surface vessels, and have a thickened marginal glial layer. Reproduced from Okada et al. (2009) with permission from Springer Nature [65].

On the other hand, a conceptual advance was provided by the cell–vessel architecture model proposed by us [18,64,65]. Based on anatomical and physiological observations in the ventrolateral medulla, this model proposed that glia-like cells strategically positioned around penetrating arterioles near the ventral medullary surface function as intrinsically chemosensitive elements. The model emphasized the intimate anatomical relationship among blood vessels, glial-like cells, and respiratory neurons, suggesting that chemosensory information may be detected initially by specialized glial elements and subsequently transmitted to neuronal respiratory networks (Figure 4). In the chemosensitive medullary region, close anatomical relationships between astrocytes and neurons, i.e., numerous astrocytic processes surrounding the somata of Phox2b-expressing RTN neurons, were reported [66].

**Figure 5:**
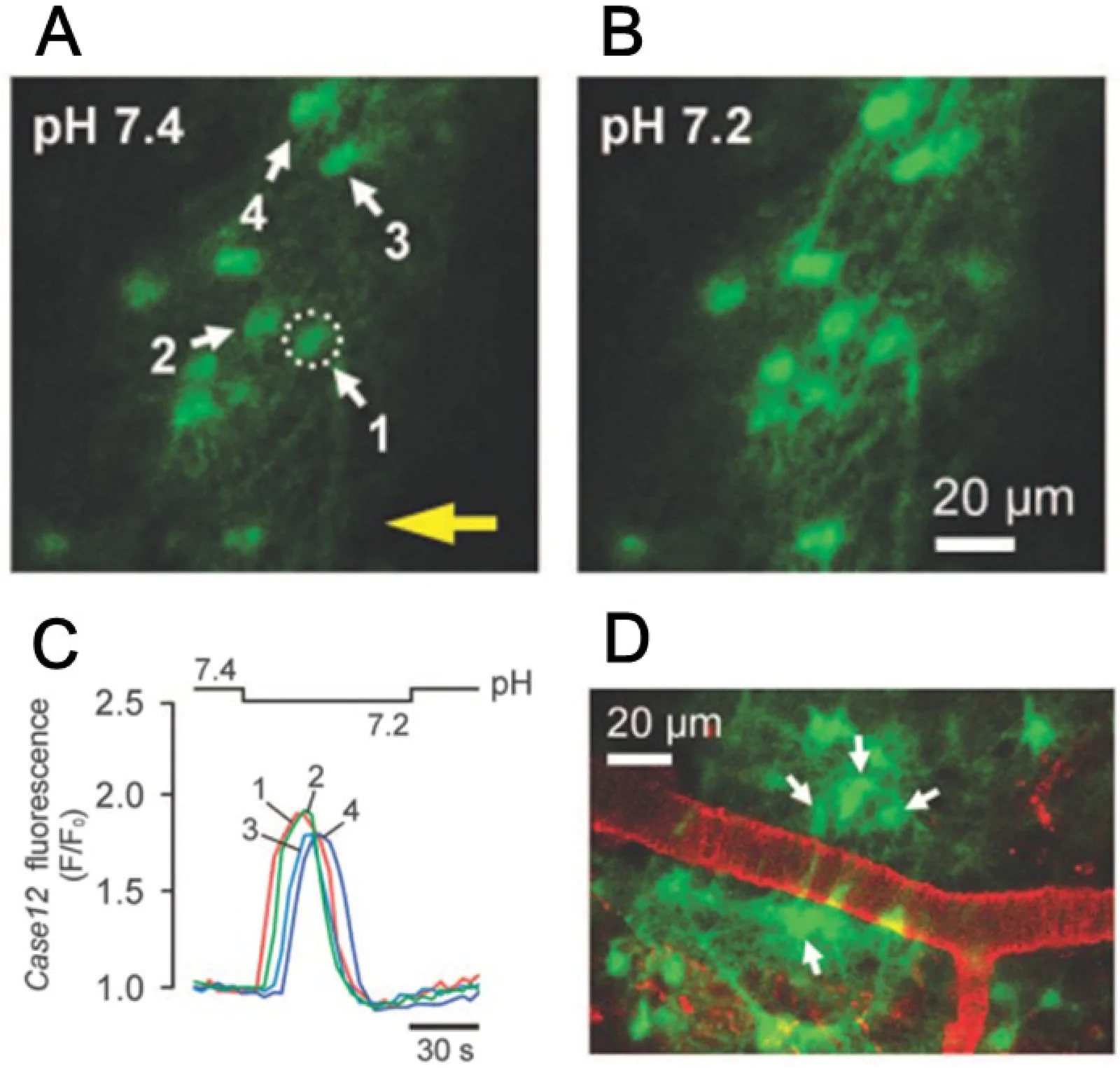
Exquisitely pH-sensitive astrocytes near the ventral medullary surface of the rat in vitro. Genetically encoded fluorescent sensor Case12 was expressed in astrocytes by using an adenoviral vector with an enhanced shortened glial fibrillary acidic protein (GFAP) promoter. (**A**). Astrocytes identified by means of Case12 fluorescence under control pH condition (pH 7.4). The yellow arrow shows the direction of superfusate flow in the chamber. (**B**). Astrocytes in the reduced-pH condition (pH 7.2). (**C**). Time course of Case12 fluorescence in cells 1–4 in response to pH changes. Acidification induced rapid increases in [Ca^2+^]i, as determined by changes in Case12 fluorescence. The circle (cell 1) indicates an astrocyte responding first to extracellular acidification. (**D**). Spatial relationship between pH-sensitive astrocytes identified by Case12 (green) and small vessels visualized with lectin (red) on the ventral surface of the rat medulla. Many pH-sensitive astrocytes, indicated by white arrows, were located adjacent to the vasculature. Reproduced from Gourine et al. (2010) with permission from The American Association for the Advancement of Science [67].

Our framework anticipated several concepts that later emerged from studies demonstrating astrocytic CO_2_/H^+^ sensitivity and gliotransmitter-mediated signaling. Evidence for astrocytic chemoreception was subsequently provided by Gourine and colleagues. Using calcium imaging, they demonstrated that physiological acidification evokes robust intracellular Ca^2+^ responses in ventral medullary astrocytes, leading to ATP release, activation of RTN chemoreceptor neurons, and stimulation of breathing (Figure 5) [67]. Furthermore, optogenetic activation of astrocytes designed to mimic hypercapnia-induced signaling elicited respiratory stimulation through a purinergic mechanism in vivo [68]. These observations strongly supported the hypothesis that astrocytes are not merely passive supporters of neuronal chemoreception but actively participate in the detection and transduction of chemosensory signals.

Aside from respiratory CO_2_/H^+^ chemoreception, medullary astrocytes have been postulated to have two other functions in respiratory control: (1) amplifying the responses of intrinsic chemosensitive neurons through feedforward signaling via gliotransmitters and (2) recruiting a greater number of non-intrinsically chemosensitive neurons to participate in the response to hypercapnia [49,69].

Astrocytes outside the medulla also play a role in central respiratory chemosensitivity. Rosatti et al. (2025) reported that lateral hypothalamic astrocytes contribute to the hypercapnic ventilatory chemoreflex in a light-dark cycle-dependent manner, i.e., during the dark-active phase, in unanesthetized rats [70].

Those accumulated experimental findings suggest a progressive shift in our understanding of central respiratory chemoreception. Rather than functioning solely as metabolic support cells or modulators of neuronal activity, astrocytes may themselves act as chemosensory elements within central respiratory networks. Although the relative contributions of astrocytic and neuronal chemoreception remain actively debated [71], it is increasingly recognized that central respiratory chemoreception emerges from dynamic interactions among neurons, astrocytes, and the local vascular environment.

### 4.2. Candidate Mechanisms of Astrocytic CO_2_/H^+^ Chemosensitivity

**Figure 6:**
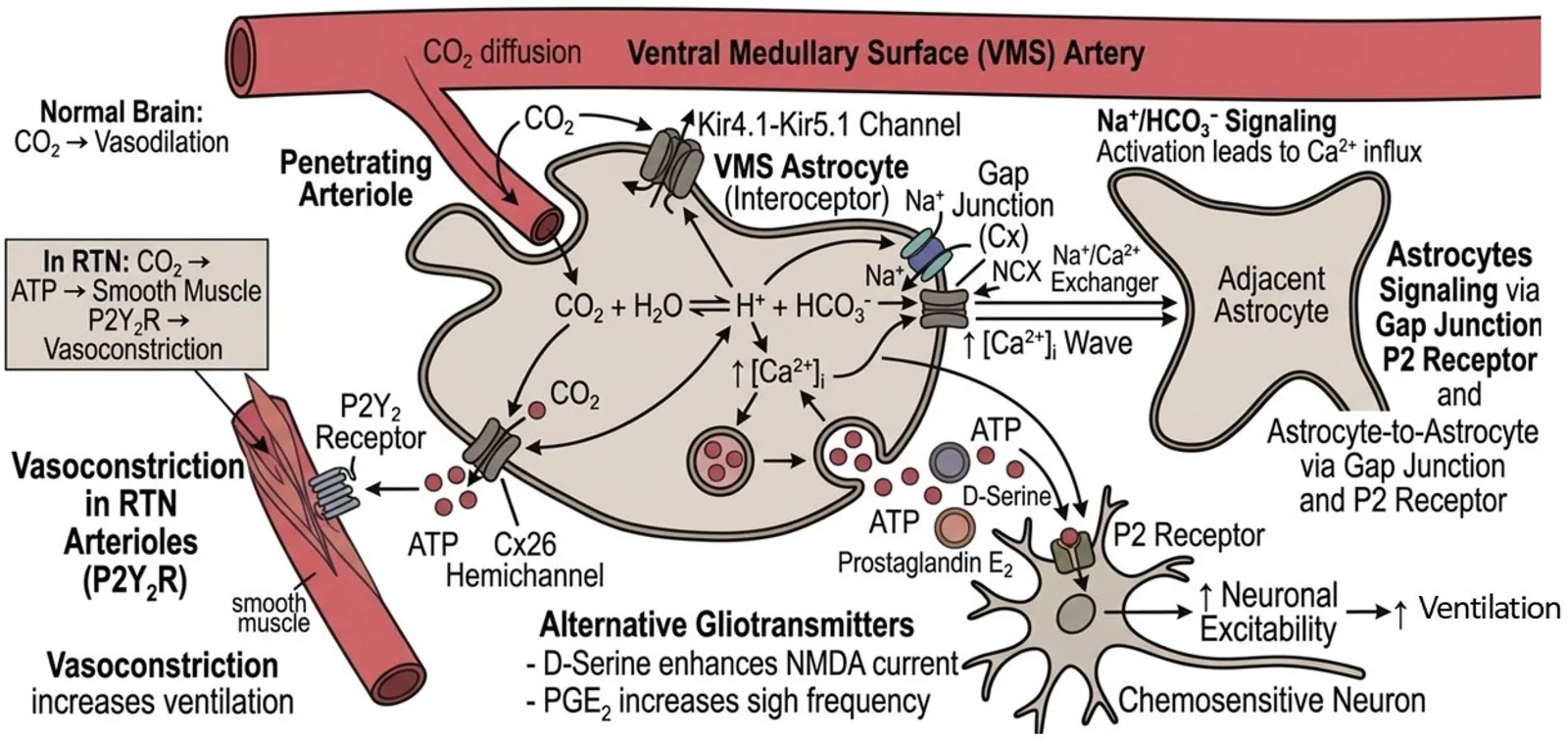
Proposed mechanisms of astrocytic CO_2_/H^+^ sensing in the ventral medullary surface region. Chemosensitive astrocytes are located close to surface vessels and sense CO_2_ diffusing out of the vessels. CO_2_ and reduced-pH promote ATP release from astrocytes through connexin-based channels, including Cx26 hemichannel, and vesicular ATP release mechanisms. CO_2_ and reduced-pH close inward rectifying potassium channels Kir4.1-Kir5.1, depolarize astrocytes, and thereby promote ATP release. ↑ sign indicates augmentation or an increase. For details, see the text.

Although substantial evidence supports a role for astrocytes in central respiratory chemoreception, the molecular mechanisms by which astrocytes detect changes in CO_2_ and pH remain incompletely understood. Current evidence suggests that astrocytic chemosensitivity is not mediated by a single signaling pathway but rather emerges from the integration of multiple molecular mechanisms [54,72,73,74,75,76]. Several candidate mechanisms have been proposed (Figure 6).

#### 4.2.1. ATP-Mediated Chemosensory Signaling

Among the proposed mechanisms, ATP-mediated signaling has received the strongest experimental support. Gourine et al. (2010) demonstrated that physiological acidification evokes robust intracellular Ca^2+^ elevations in ventral medullary astrocytes, resulting in ATP release and activation of neighboring chemoreceptor neurons [67]. Optogenetic stimulation of astrocytes mimicking hypercapnia-induced Ca^2+^ signaling similarly enhanced respiratory activity through a purinergic mechanism [68]. These observations established ATP as a principal gliotransmitter linking astrocytic chemosensitivity to respiratory network activation. Kasymov et al. (2013) reported that brainstem astrocytes respond to acidification by enhancing the exocytosis of ATP-containing vesicular compartments [77]. Conversely, cortical astrocytes exhibit a higher basal rate of vesicular fusion at pH 7.4, which remains unaffected by extracellular acidification [77]. These findings indicate that brainstem astrocytes in the chemosensory area possess a distinct functional sensitivity to pH alterations compared to their cortical counterparts.

#### 4.2.2. Direct CO_2_ Sensing Through Connexin26 Hemichannels

An attractive mechanism proposes that astrocytes directly detect CO_2_ through connexin26 (Cx26) hemichannels. Unlike conventional pH-dependent signaling pathways, Cx26 appears capable of sensing molecular CO_2_ directly. Elevated PCO_2_ promotes opening of Cx26 hemichannels, leading to ATP release and activation of respiratory circuits [78,79,80,81,82]. This mechanism provides one of the clearest examples of direct CO_2_ sensing in the mammalian nervous system.

#### 4.2.3. Kir4.1/Kir5.1 Potassium Channel-Mediated Chemosensitivity

A second major mechanism involves inwardly rectifying potassium channels expressed by astrocytes. Hypercapnia inhibits Kir4.1/Kir5.1 channel activity, resulting in membrane depolarization and ATP release. The released ATP subsequently enhances RTN chemoreceptor neuron activity through purinergic signaling pathways [73,74,75,83,84]. This model integrates changes in extracellular CO_2_ with astrocytic membrane excitability and gliotransmitter release.

#### 4.2.4. Na^+^/HCO_3_^−^ Transporter-Dependent Signaling

Acidification also activates the Na^+^/HCO_3_^−^ cotransporter, leading to intracellular Na^+^ accumulation and reversal of Na^+^/Ca^2+^ exchange activity. The resulting Ca^2+^ influx can trigger intracellular signaling cascades and gliotransmitter release [85]. This mechanism provides a potential link between intracellular acid-base regulation and astrocytic chemosensory signaling. However, recent evidence suggesting that Na^+^/HCO_3_^−^ cotransport is dispensable for respiratory chemosensitivity indicates that its physiological significance remains controversial [86].

#### 4.2.5. Alternative Gliotransmitters: D-Serine and Prostaglandin E_2_

Although ATP is considered the dominant gliotransmitter involved in respiratory chemoreception, other signaling molecules may contribute. Astrocytes within the caudal medulla synthesize and release D-serine in response to elevated CO_2_ levels, thereby enhancing NMDA receptor-mediated respiratory excitation [87,88]. In addition, pFRG/RTN astrocytes release prostaglandin E_2_ during hypercapnic stimulation, increasing sigh frequency and inspiratory depth [38].

#### 4.2.6. Ventilatory Augmentation Through Astrocyte-Mediated CO_2_-Dependent Vasoconstriction in the RTN

Blood flow and its distribution in brain regions, including the medullary chemosensitive areas, are highly sensitive to changes in the regional tissue CO_2_ level. Increased blood flow from CO_2_-induced vasodilation enhances the removal of metabolic CO_2_. Consequently, the extracellular pH rises, acting as a buffer that limits the CO_2_-driven increase in ventilation [83,89,90]. Although the mechanisms of CO_2_-induced blood flow changes have not been fully elucidated, astrocytes are involved in CO_2_-dependent vasodilation in wide brain regions [91,92]. Not in the RTN, though, where CO_2_ uniquely induces vasoconstriction. When the tissue CO_2_ level increases, astrocytes release ATP, which acts at P2Y_2_ receptors in smooth muscle cells of RTN arterioles, causing vasoconstriction and contributing to ventilatory augmentation [93,94].

These observations suggest that astrocytes can shape multiple components of respiratory behavior through the release of distinct gliotransmitters. Available evidence supports a model in which astrocytic chemosensitivity emerges from the interaction of multiple molecular pathways rather than a single dedicated CO_2_ sensor. Determining the relative contribution of these mechanisms under physiological conditions remains a major challenge for future studies.

## 5. Role of Astrocytes in Hypoxic Respiratory Responses

For many years, respiratory responses to hypoxia were thought to depend exclusively on peripheral chemoreceptors, particularly carotid bodies. However, accumulating evidence indicates that oxygen-sensing mechanisms are also present within the central nervous system and contribute to the regulation of breathing during hypoxic challenges [11,81,95,96]. Among the candidate central oxygen sensors, astrocytes have emerged as important cellular elements capable of detecting decreases in tissue oxygen availability and modulating respiratory network activity. It must be noted that hypoxic respiratory responsive mechanisms could be different among acute moderate hypoxia, acute severe hypoxia, chronic intermittent hypoxia and acclimatization to hypoxia because they are biologically distinct states.

### 5.1. Acute Moderate Hypoxia

A major advance in this field was provided by Angelova et al. (2015), who demonstrated that astrocytes are intrinsically sensitive to physiologically relevant acute reduction in PO_2_ [97]. Hypoxia suppresses mitochondrial respiration in astrocytes, resulting in mitochondrial depolarization, generation of reactive oxygen species, lipid peroxidation, activation of phospholipase C, and IP_3_-dependent release of Ca^2+^ from intracellular stores. The resulting increase in intracellular Ca^2+^ triggers exocytosis of ATP-containing vesicles and ATP release into the extracellular space [97,98]. These findings established a mechanistic framework linking oxygen sensing in astrocytes to gliotransmitter-mediated modulation of respiratory circuits.

ATP released from astrocytes appears to play a central role in the respiratory response to hypoxia. In the preBötC, astrocyte-derived ATP acts on P2Y_1_ receptors expressed by both neurons and astrocytes, promoting further intracellular Ca^2+^ signaling and enhancing respiratory activity [51,99]. This positive-feedback mechanism is thought to counteract secondary hypoxic respiratory depression and help maintain respiratory drive during oxygen deprivation.

Evidence for astrocytic participation in hypoxic responses has also been obtained from other brainstem regions. Kato et al. (2025) reported that hypoxia increased Fos expression in astrocytes located in the rostral raphe pallidus, whereas serotonergic neurons in the same region showed no significant activation [100]. Electrical stimulation of this region increased respiratory frequency in anesthetized rats, suggesting that raphe astrocytes contribute directly to hypoxia-induced respiratory excitation [100].

**Figure 7:**
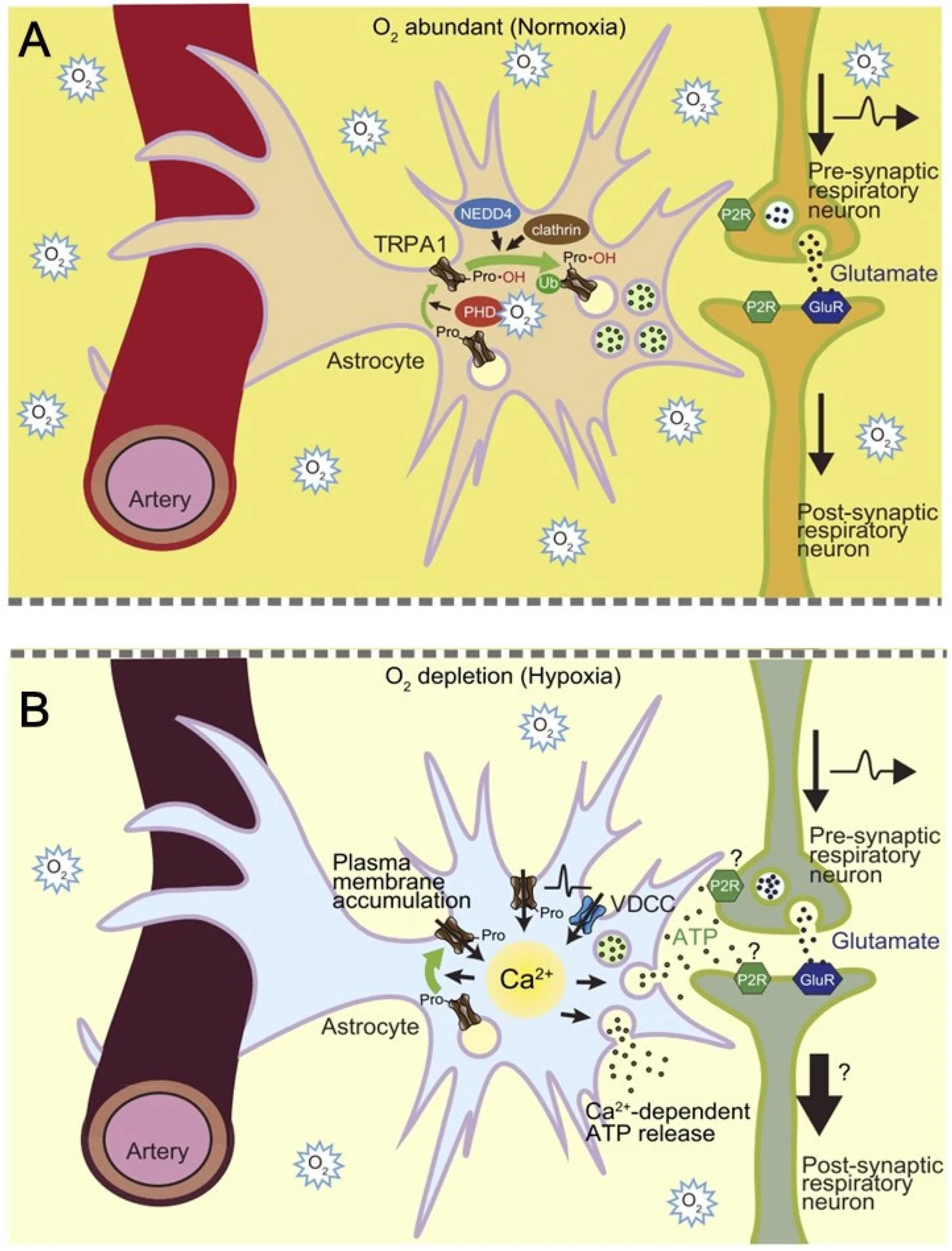
A model of molecular mechanisms underlying hypoxia sensing in TRPA1 of pFRG/RTN astrocytes. (**A**). O_2_-abundant conditions, such as normoxia, hyperoxia, and hypercapnia, lead to the prolyl hydroxylation of TRPA1 by prolyl hydroxylase (PHD). The hydroxylated TRPA1 at the plasma membrane is tagged with ubiquitin (Ub) by NEDD4-1 (NEDD4) and then removed from the plasma membrane through clathrin-dependent endocytosis to early endosomes. (**B**). O_2_ deprivation diminishes PHD activity and relieves TRPA1 from the prolyl hydroxylation, leading to plasma membrane accumulation of TRPA1. The Ca^2+^ influx via TRPA1 and its downstream voltage-dependent calcium channels (VDCCs) accelerate ATP release from astrocytes, modulating respiratory activity. Reproduced from Uchiyama et al. (2020) with permission from Elsevier [102].

Recent evidence has identified additional molecular pathways through which astrocytes may detect moderate hypoxia. Our studies demonstrated that oxygen negatively regulates transient receptor potential ankyrin 1 (TRPA1) channels through oxygen-dependent proline hydroxylation and subsequent protein internalization. During hypoxia, suppression of this regulatory mechanism increases TRPA1 channel expression at the plasma membrane, resulting in Ca^2+^ influx and ATP release from pFRG/RTN astrocytes (Figure 7) [101,102]. Activation of this pathway enhances respiratory activity and represents a novel mechanism by which astrocytes contribute to central oxygen sensing.

### 5.2. Acute Severe Hypoxia

Astrocytes may also protect against secondary hypoxic respiratory depression through mechanisms extending beyond direct modulation of respiratory neuronal networks. We previously demonstrated that astrocytic activation contributes to the maintenance of arousal and consciousness during acute severe hypoxia, thereby preserving consciousness-dependent respiratory drive [103,104]. Consistent with this concept, Czeisler et al. (2019) identified a subpopulation of RTN astrocytes derived from the embryonic PHOX2B progenitor domain that is selectively sensitive to reduction in oxygen tension [105]. Ablation of these astrocytes exacerbated secondary hypoxic respiratory depression, indicating an important protective role during severe hypoxic stress.

### 5.3. Chronic Intermittent Hypoxia and Acclimatization to Hypoxia

Although responses to chronic intermittent hypoxia and acclimatization to hypoxia are physiologically distinct, they have been often studied together. Therefore, here we review these issues together. The role of astrocytes within the NTS in response to chronic intermittent hypoxia appears to be complex. Costa et al. (2013) reported that selective astrocytic inhibition in the NTS had little effect on baseline respiratory activity or on adaptations induced by chronic intermittent hypoxia [106]. Tadmouri et al. (2014) demonstrated that astrocytes are activated during the early phase of hypoxic exposure and contribute to neuronal responses during the first hours of acclimatization, whereas microglia become increasingly involved during later stages through astrocyte–microglia interactions [107]. More recent studies have further highlighted the dynamic role of NTS astrocytes. Bazilio et al. (2024) showed that astrocytes regulate baseline respiratory frequency through glutamatergic and purinergic signaling mechanisms, although this influence is diminished after sustained hypoxic exposure [108]. Similarly, Pereyra et al. (2025) demonstrated that chronic intermittent hypoxia induces an early astrocytic response followed by prolonged microglial activation in the NTS, accompanied by respiratory instability and enhanced hypoxic ventilatory responses [109]. However, Luz et al. (2026) reported that sustained hypoxia increased postsynaptic neuronal excitability without detectable alterations in astrocyte-related signaling mechanisms [110]. These apparently conflicting findings suggest that the contribution of NTS astrocytes may depend on the duration, severity, and pattern of hypoxic exposure.

### 5.4. Summary of Astrocytic Responses to Various Modalities of Hypoxia

Based on these observations, we propose that astrocytes participate actively in central sensing of and respiratory adaptation to various modalities of hypoxia. Through mitochondrial oxygen-sensing pathways, TRPA1-dependent mechanisms, ATP-mediated gliotransmission, and interactions with neurons and microglia, astrocytes contribute to both acute hypoxic ventilatory responses and longer-term acclimatization processes. Although peripheral chemoreceptors remain the primary sensors of arterial oxygen tension, astrocytes are increasingly recognized as important central components of the integrated respiratory response to hypoxia.

## 6. Role of Astrocytes in Respiratory Plasticity

Recent advances in neuroscience have revealed that astrocytes are active participants in learning, memory formation, and neural plasticity. Through bidirectional communication with neurons, astrocytes regulate synaptic transmission, influence network excitability, and contribute to long-term information storage [111,112,113,114,115,116]. Astrocytes have also been implicated in long-lasting autonomic adaptations, including persistent stress-induced elevations in arterial blood pressure [117]. These observations suggest that astrocytes may represent a common cellular substrate underlying neural plasticity across multiple physiological systems.

**Figure 8:**
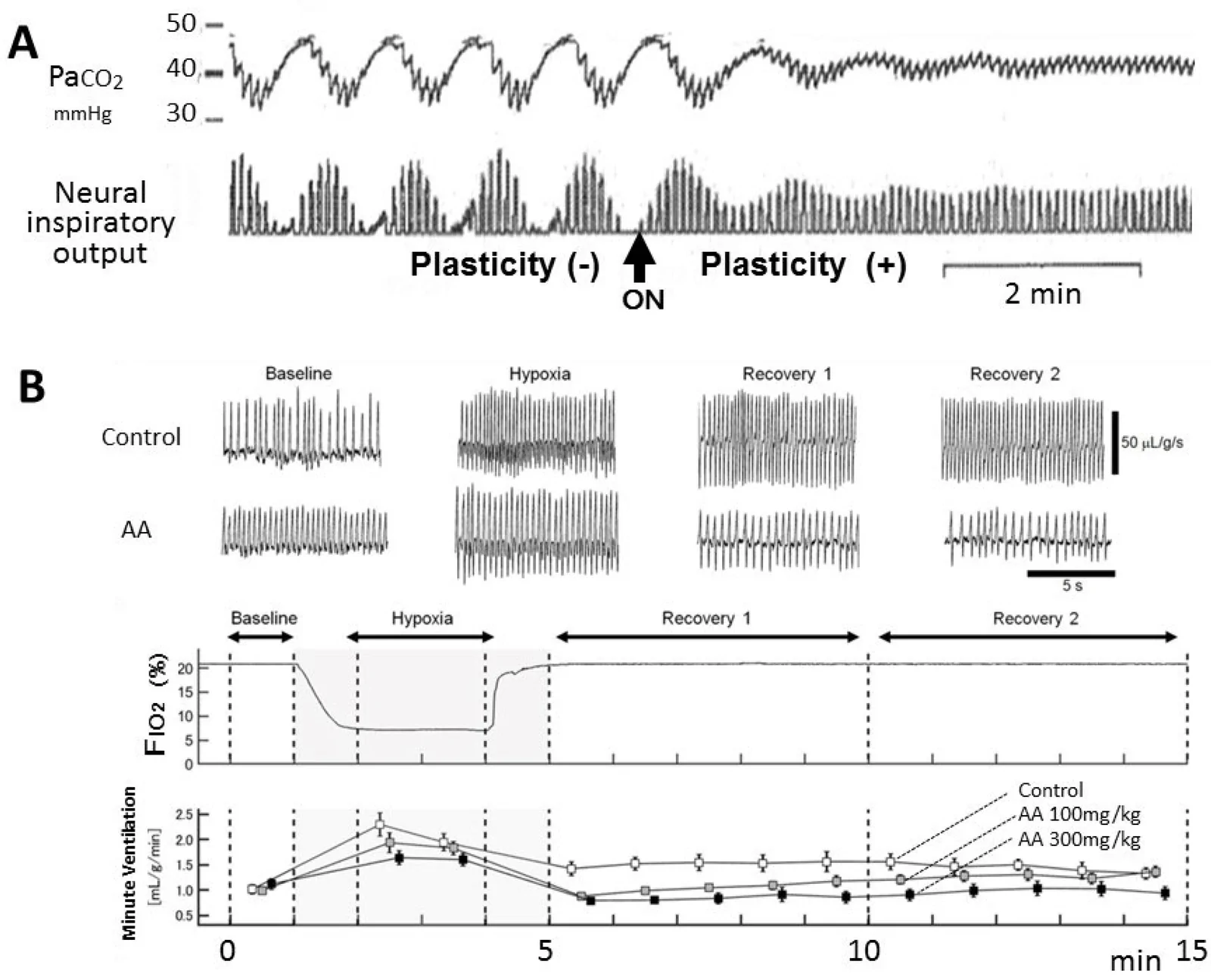
(**A**). Significance of respiratory plasticity (computer simulation). When respiratory plasticity is incorporated, periodic breathing is reduced and respiratory output becomes more stable. Adapted from Eldridge (1996) with permission from Springer Nature [118]. (**B**). Effects of arundic acid (AA) on hypoxic ventilatory responses in mice by whole-body plethysmography. Two top traces: Representative recordings of respiratory flow (inspiration upward) in mice without and with 300 mg/kg AA in room air (Baseline), 7% Hypoxia, Recovery phase 1 (first 5 min), and Recovery phase 2 (second 5 min). Middle trace: Time profile of chamber oxygen changes. Bottom trace: Time series data for minute ventilation in the control (no AA), 100 mg/kg, and 300 mg/kg AA dose conditions. AA did not affect baseline minute ventilation but tended to attenuate the acute hypoxic hyperventilation and notably inhibited post-hypoxic persistent respiratory augmentation (PHRA). Adapted from Fukushi et al. (2021) under the terms of Creative Commons CC BY 4.0 [119].

Respiratory plasticity refers to persistent changes in respiratory responsiveness that are induced by previous activity of the respiratory control system. In this context, respiratory responses to external stimuli or internal environmental challenges are influenced by preceding respiratory activity, allowing adaptive modifications of breathing behavior. Respiratory plasticity is considered essential for maintaining the stability of respiratory control and preventing respiratory instability (Figure 8A) [118].

One well-characterized example of respiratory plasticity is post-hypoxic respiratory augmentation (PHRA) [119]. Acute hypoxia increases ventilation through activation of peripheral and central respiratory pathways. Following restoration of normoxia, ventilation gradually declines but often remains elevated above baseline levels for a while. This sustained enhancement of ventilation is thought to stabilize breathing and reduce the likelihood of periodic breathing or recurrent respiratory instability. Although the neuronal mechanisms underlying respiratory plasticity have been extensively investigated [120,121], the potential contribution of astrocytes has received comparatively little attention. Given the established role of astrocytes in other forms of neural plasticity, we hypothesized that astrocytic signaling may also participate in the generation and maintenance of respiratory plasticity. To test this hypothesis, we analyzed hypoxic ventilatory responses in mice using whole-body plethysmography [119]. Animals were exposed sequentially to room air, a hypoxic gas mixture (7% O_2_, 93% N_2_) for 2 min, and subsequently room air. Experiments were performed before and after administration of low and high doses of arundic acid (ONO-2506), a compound that suppresses astrocytic activation by inhibiting production of the astrocytic protein S100β [103,104,122]. Pharmacological inhibition of astrocytic activation markedly attenuated PHRA. Furthermore, administration of a higher dose of arundic acid reduced post-hypoxic ventilation to levels below the pre-hypoxic baseline. These findings indicate that astrocytes contribute to the maintenance of post-hypoxic respiratory facilitation and suggest that astrocytic signaling participates in respiratory plasticity (Figure 8B). Astrocytes may be involved not only in the moment-to-moment regulation of breathing but also in adaptive changes in respiratory control that persist beyond the initiating stimulus. These findings extend the emerging concept that astrocytes are integral components of neural plasticity mechanisms throughout the central nervous system. The involvement of astrocytes in respiratory plasticity must be confirmed in experiments with another study design. The mechanisms through which astrocytes influence respiratory plasticity remain incompletely understood. Future studies will be required to determine how astrocytic signaling interacts with established neuronal mechanisms of respiratory plasticity.

## 7. Involvement of Astrocytes in the Pathophysiology of Respiratory Control Disorders

Growing evidence indicates that astrocytes are not only essential components of normal respiratory control networks but also contribute to the pathogenesis of several neurological disorders associated with abnormal breathing. Alterations in astrocytic development, signaling, metabolism, or neuron–astrocyte communication may impair respiratory rhythm generation, chemoreception, hypoxic responsiveness, and respiratory plasticity. The following examples illustrate the emerging role of astrocytic dysfunction in respiratory control disorders.

### 7.1. Brainstem Astrocytoma

Brainstem tumors involving the pons or medulla are frequently associated with respiratory disturbances, including periodic breathing, ataxic breathing, sleep apnea, hypoventilation, and respiratory arrest [123,124]. In rare cases, however, astrocytomas arising within the brainstem are accompanied by central neurogenic hyperventilation [125,126,127,128].

The mechanisms responsible for this severe and treatment-resistant hyperventilation remain poorly understood. Proposed explanations include local tissue acidosis in the brainstem chemosensitive region, disruption of the ponto-medullary descending respiration-inhibitory pathway, or mechanical stimulation of the medullary respiratory neuron network by invading tumor cells. However, it is hard to imagine that those mechanisms induce extremely severe hyperventilation. On the other hand, it is probable that the cause is an uncontrolled excess release of ATP from abnormally proliferated astrocytes. It is conceivable that ATP released from tumor cells binds to P2Y_1_ receptors in the preBötC neurons to stimulate the medullary respiratory network [51,99].

We hypothesize and propose that inhibition of the local ATP action with a P2Y_1_ antagonist, e.g., by selective intra-arterial injection of MRS2179 [99], or blockade of vesicular exocytosis of ATP from the astrocytoma using an adenoviral vector to specifically express tetanus toxin light chain in astrocytes may be effective in alleviating treatment-resistant hyperventilation [51]. Although this is speculative and untested treatment, central neurogenic hyperventilation accompanied by human brain astrocytoma has been increasingly reported, and is resistant to conventional pharmacological treatment and devastating for the patients [128], our proposal may be worth investigating. Future studies examining purinergic signaling within astrocytomas may provide new insights into the pathogenesis of central neurogenic hyperventilation and could potentially identify novel therapeutic targets for this otherwise difficult-to-treat condition.

### 7.2. Rett Syndrome

Rett syndrome is a severe neurodevelopmental disorder caused by mutations in the X-linked gene *MECP2*, which encodes methyl-CpG-binding protein 2 (MeCP2), a multifunctional regulator of gene expression. Affected individuals commonly exhibit seizures, motor dysfunction, irregular breathing, rapid shallow breathing, reduced ventilatory responsiveness to CO_2_, apnea, and episodes of hyperventilation [129,130].

Importantly, MeCP2 is expressed not only in neurons but also in astrocytes. Indeed, astrocytes in Rett syndrome accompany structural and functional impairments. MECP2 deficient astrocytes secrete abnormal factors impairing neuronal growth and synaptic function, and show dysregulated calcium signalling, disrupted glutamate and potassium homeostasis, and increased inflammatory responses [131]. Studies using *Mecp2*-deficient mice demonstrated that selective restoration of MeCP2 expression in astrocytes substantially improves respiratory abnormalities and rescues normal breathing patterns [132,133]. These findings indicate that astrocytic dysfunction contributes directly to the respiratory phenotype of Rett syndrome. Deficient astrocytic CO_2_/H^+^ sensing and impaired astrocyte–neuron communication have been proposed as mechanisms underlying the reduced ventilatory chemosensitivity observed in Rett syndrome. Thus, Rett syndrome provides supporting evidence that astrocytic abnormalities can profoundly disrupt respiratory control.

### 7.3. Sudden Infant Death Syndrome (SIDS)

The pathogenesis of sudden infant death syndrome (SIDS) remains incompletely understood, but abnormalities of brainstem respiratory control have long been implicated. Several investigators have proposed that developmental defects affecting astrocytes may contribute to SIDS [134]. Histological studies revealed immature astrocytic morphology within the brainstems of infants who died from SIDS, suggesting delayed maturation of central nervous system structures involved in respiratory regulation [135]. These observations support the concept that SIDS may represent a developmental disorder of brainstem function.

Other neuropathological studies have produced conflicting results. Pamphlett and Treloar (1996) found no significant differences in astrocytosis within the hypoglossal nucleus between SIDS and control infants, suggesting that severe antecedent hypoxic–ischemic injury was unlikely [136]. In contrast, Biondo et al. (2004) reported increased reactive astrocytosis in the NTS, potentially reflecting previous hypoxic injury within respiratory control pathways [137]. Although the available evidence remains inconclusive, these observations suggest that abnormal astrocyte development or function may contribute to respiratory instability in susceptible infants.

### 7.4. Sudden Unexpected Death in Epilepsy (SUDEP)

Respiratory failure occurring during or immediately after seizures is considered a major contributor to sudden unexpected death in epilepsy (SUDEP). Recent evidence suggests that astrocytic dysfunction may influence susceptibility to fatal respiratory arrest. Patodia et al. (2019) reported reduced numbers of vimentin-positive astrocytes in the ventrolateral medulla and decreased expression of the astrocytic gap-junction protein connexin43 within the median raphe of SUDEP victims [138]. These findings suggest impaired astrocyte–neuron communication and compromised respiratory responsiveness.

In contrast, our experimental studies demonstrated that pharmacological suppression of astrocytic activation using arundic acid delayed the onset of severe hypoxia-induced seizures and respiratory arrest in mice [104]. These findings raise the possibility that excessive astrocytic activation during profound hypoxia may promote seizure generation, seizure-induced respiratory arrest, and death.

Taken together, current evidence suggests that both insufficient and excessive astrocytic activity may adversely affect respiratory control under pathological conditions. Further investigation of astrocyte-dependent mechanisms may improve our understanding of SUDEP pathogenesis and identify novel strategies for preventing seizure-associated respiratory failure.

### 7.5. Concluding Remarks on Astrocyte-Related Respiratory Disorders

Although the underlying mechanisms differ among individual disorders, a common theme emerges: disruption of normal astrocytic signaling can impair respiratory network function at multiple levels, including rhythm generation, chemoreception, hypoxic responsiveness, and respiratory plasticity. Increasing recognition of astrocytes as active participants in respiratory control therefore provides a new framework for understanding the pathophysiology of respiratory disorders and may reveal novel therapeutic opportunities for diseases that are currently difficult to treat.

## 8. Methodological Limitations

### 8.1. Limitations in Experimental Models

In studies on the role of astrocytes in respiratory control, various kinds of experimental models have been utilized. Each model has its distinct strengths and limitations, which are summarized in Table 1. When evaluating the experimental results, these limitations should always be kept in mind.

### 8.2. Limitations in Astrocyte-Specific Manipulation

A major challenge in interpreting the current literature is the limited specificity of many experimental approaches used to manipulate or identify astrocytes. Pharmacological agents such as fluorocitrate, fluoroacetate, methionine sulfoximine, and arundic acid have provided valuable insights into astrocytic function; however, none of these agents is exclusively selective for astrocytes, and off-target effects cannot be completely excluded. Likewise, immunohistochemical markers including GFAP and S100β are widely used to identify astrocytes but do not uniformly label all astrocyte populations and may also be expressed by other cell types under certain conditions (Table 2). Therefore, conclusions regarding astrocyte-specific mechanisms should be interpreted with caution, particularly when based on a single experimental approach.

To overcome these limitations, future studies should combine astrocyte-specific genetic approaches with functional analyses. Chemogenetic and optogenetic manipulation using astrocyte-specific Cre driver lines, such as Aldh1L1-CreERT2 mice, together with calcium imaging, fiber photometry, or single-cell transcriptomics, will provide substantially greater cell-type specificity than conventional pharmacological approaches [139]. These techniques will facilitate a more precise understanding of the role of astrocytes in central respiratory control.

## 9. Future Perspectives

The traditional view of astrocytes as passive support cells has undergone a profound transformation over the past two decades. Accumulating evidence now indicates that astrocytes play modulatory roles in close communication with neurons in various aspects of respiratory control, including respiratory rhythm and pattern generation, central chemoreception, hypoxic responsiveness, respiratory plasticity, and the pathophysiology of respiratory disorders. Nevertheless, many fundamental questions remain unresolved.

One of the most important challenges is to determine the precise contribution of astrocytes to respiratory rhythmogenesis. Although substantial evidence supports a modulatory role for astrocytes within respiratory networks, it remains unclear whether specific astrocyte populations directly participate in rhythm generation or primarily regulate neuronal rhythmogenic mechanisms. Similarly, the relative contributions of neuronal and astrocytic chemosensory pathways to central CO_2_/H^+^ detection remain an area of active investigation.

Another important goal will be to identify the molecular mechanisms underlying astrocytic CO_2_/H^+^ and oxygen sensing. Recent studies have implicated ATP signaling, connexin26 hemichannels, Kir4.1/Kir5.1 potassium channels, TRPA1 channels, and other signaling pathways. However, how these mechanisms interact under physiological conditions and whether they operate in distinct astrocyte subpopulations remain largely unknown.

The increasing recognition of astrocyte heterogeneity also presents new opportunities for future research. Astrocytes located within the preBötC, pFRG/RTN, NTS, raphe nuclei, and spinal respiratory motor regions likely possess distinct molecular signatures and physiological functions. Advances in single-cell transcriptomics, spatial transcriptomics, and cell-type-specific genetic manipulation are expected to provide unprecedented insights into the functional specialization of respiratory astrocytes.

From a clinical perspective, astrocytes are emerging as promising therapeutic targets for respiratory disorders. Abnormal astrocytic signaling has been implicated in brainstem astrocytoma, Rett syndrome, SIDS, SUDEP, and other disorders associated with respiratory dysfunction. A better understanding of astrocyte–neuron communication may therefore facilitate the development of novel therapeutic approaches aimed at restoring respiratory network stability and improving respiratory outcomes.

Future investigations combining advanced imaging techniques, optogenetics, chemogenetics, molecular genetics, and translational studies in humans will undoubtedly expand our understanding of astrocyte biology in respiratory control. Such efforts are likely to reshape current concepts of respiratory regulation and may ultimately lead to innovative therapies for respiratory control disorders that remain difficult to treat.

In conclusion, astrocytes should no longer be regarded merely as supporting elements of respiratory neural circuits. Rather, they are increasingly recognized as integral components of the respiratory control system whose influence extends from moment-to-moment regulation of breathing to long-term adaptive and pathological processes. Continued exploration of astrocyte function is expected to provide important new insights into both the physiology and pathophysiology of respiratory control.

## Figures and Tables

**Table 1:** Strengths and limitations of experimental models commonly used to investigate astrocyte function in respiratory control.

Model	Primary Use	Strengths	Major Limitations
**Neonatal brain slices**	Cellular mechanisms	Excellent experimental control	Immature networks; lacks systemic inputs and adult physiology
**Anesthetized animals**	Integrated physiology	Preserves intact neural networks	Anesthesia alters neuronal, astrocytic, and respiratory functions
**Isolated brainstem preparation**	Respiratory rhythm generation	Stable brainstem preparation	Lacks forebrain and peripheral inputs
**Working heart–brainstem preparation**	Cardiorespiratory integration	Near-physiological brainstem function without anesthesia	No forebrain regulation; technically demanding
**Pharmacological inhibition**	Functional manipulation	Simple and widely applicable	Limited astrocyte specificity; possible off-target effects
**Fiber photometry**	In vivo population activity	Long-term recording in behaving animals	Correlative only; limited spatial resolution
**Confocal or two-photon calcium imaging**	Cellular Ca^2+^ signaling	High spatial resolution	Usually ex vivo; limited physiological context

**Table 2:** Limitations of commonly used approaches for investigating astrocytic function.

Method	Major Limitations
**Fluorocitrate**	Although relatively selective for astrocytes, fluorocitrate is not completely astrocyte specific. At higher concentrations, it may also affect neuronal function. Because it inhibits cellular metabolism, interpretation of its physiological effects is limited.
**Fluoroacetate**	Like fluorocitrate, fluoroacetate inhibits cellular metabolism and lacks absolute specificity for astrocytes. Off-target effects cannot be excluded.
**Methionine sulfoximine**	Inhibits glutamine synthetase, a predominantly astrocytic enzyme, but does not selectively manipulate all astrocytes.
**Arundic acid (ONO-2506)**	Frequently described as an astrocyte inhibitor; however, its precise mechanism of action remains incompletely understood.
**GFAP**	Widely used astrocyte marker, but preferentially labels reactive astrocytes. Its expression is low or absent in immature astrocytes and in subsets of mature astrocytes.
**S100β**	Commonly used as an astrocyte marker, but is also expressed in other cell types, including oligodendrocytes under certain conditions; therefore, it is not exclusively astrocyte-specific.

## Data Availability

No new data were created or analyzed in this study. Data sharing is not applicable to this article.
